# TEX9 and eIF3b functionally synergize to promote the progression of esophageal squamous cell carcinoma

**DOI:** 10.1186/s12885-019-6071-9

**Published:** 2019-09-03

**Authors:** Fengkai Xu, Shu Zhang, Zhonghe Liu, Jie Gu, Yin Li, Lin Wang, Wei Mao, Qiaoliang Zhu, Huankai Shou, Di Ge, Chunlai Lu

**Affiliations:** 1 Department of Thoracic Surgery, Zhongshan Hospital, Fudan University, Shanghai, 200032 China; 2 Liver Cancer Institute, Zhongshan Hospital, Key Laboratory of Carcinogenesis and Cancer Invasion (Ministry of Education), Fudan University, Shanghai, 200032 China

**Keywords:** Esophageal squamous cell carcinoma, Eukaryotic translation initiation factors 3 subunit b, Testis-expressed protein 9, AKT signaling pathway, Proteomics

## Abstract

**Background:**

Esophageal squamous cell carcinoma (ESCC) is one of the most frequent malignant digestive tumors around the world. We previously demonstrated that eIF3b could promote the progression of ESCC. The exact mechanisms underlying these effects remained unknown.

**Methods:**

Quantitative proteomics was applied to detect the potential targets of Eukaryotic translation initiation factor 3 subunit b (eIF3b). RT-qPCR and Western blot were performed to detect the expression of targeted gene and pathway related genes. RNA-immunoprecipitation was applied to verify the binding of eIF3b with targeted gene. Moreover, CCK-8 assay, colony-formation assay, transwell assay, flow cytometry for cell apoptosis and tumor xenograft assay were performed to analyze the regulation of the targeted gene on the bio-function of ESCC cells.

**Results:**

Quantitative proteomics data showed that Testis-expressed protein 9 (TEX9) expression was positively associated with eIF3b expression. RT-qPCR and Western blot results confirmed the quantitative proteomics data and demonstrated that TEX9 expression was positively correlated with TNM stage in ESCC. Furtherly, RNA-immunoprecipitation confirmed that eIF3b binding to TEX9 mRNA. The bio-function related assay demonstrated that TEX9 and eIF3b functionally synergized to promote the proliferation and migration, and inhibited the apoptosis of ESCC cells. In the analysis of mechanism, we revealed that TEX9 and eIF3b promoted the progression of ESCC through the activation of AKT signaling pathway.

**Conclusions:**

The synergized promoting role of TEX9 and eIF3b in the progression of ESCC may provide a novel mechanism for exploring viable therapeutic strategies for ESCC.

**Electronic supplementary material:**

The online version of this article (10.1186/s12885-019-6071-9) contains supplementary material, which is available to authorized users.

## Background

Esophageal carcinoma is one of the most frequent digestive malignant tumors around the world and ESCC is the major pathological type in China [1]. Recently, electronic endoscopy has become one of the most sensitive approaches for the diagnosis and treatment of esophageal cancer. It was reported the agents that inhibited erb-b2 receptor tyrosine kinase 2 (ERBB2 or HER2), or vascular endothelial growth factor (VEGF), including Trastuzumab, Ramucirumab and Apatinib, could increase response and survival times of ESCC patients [2]. Despite these improvements, the 5-year survival rate of ESCC patients remains below 40% [3]. Carcinogenesis mechanism of ESCC should be elucidated comprehensively to discover the applicable and effective therapeutic target.

Deregulated protein synthesis and degradation contribute to cancer genesis and progression. Eukaryotic translation initiation factors (EIFs) is a family that involves in the process of total protein synthesis. Among all the family members of eIFs, eIF3 is the largest factor, 800-kDa molecular, which consists of 13 subunits named eIF3a to eIF3m, contributing to the maintenance of 40S subunits and assembling of 43S pre-initiation complex [4]. eIF3b is aberrantly expressed or activated in different types of human cancer. For example, it promoted proliferation and inhibited apoptosis in glioblastoma cells [5]. In addition, its expression related to human bladder and prostate cancer prognosis and was required for tumor growth [5]. In previous study, we have demonstrated that eIF3b was up-regulated in ESCC and promoted the progression of ESCC [6]. To identify the mechanisms underlying these effects, especially whether through alterations in the rates of synthesis of specific proteins, quantitative proteomics was considered in this study.

Mass spectrometry-based proteomics has evolved as a powerful technology for proteome-wide detection and quantification of proteins in diseases [7]. Isobaric labelling such as TMT enable quantitative measurement in different biological samples. In this study, we analyzed and compared the proteome from eIF3b-depleted, eIF3b-overexpressed and EC109 cell by TMT technology coupled with nanoscale liquid chromatography tandem mass spectrometry (LC-MS/MS). Based on proteomics, we focused on TEX9 and found that it binded with eIF3b to promote the proliferation and migration of ESCC, inhibit cell apoptosis through the activation of AKT signaling pathway. This synergized function provides a novel mechanism for exploring viable therapeutic strategies for ESCC.

## Methods

### Tissue sample

Totally, 25 pairs of frozen human ESCC tissues and matched adjacent nontumorous tissues were obtained from Zhongshan Hospital in 2017. The tissues were stored at − 80 °C. The tumor stage was determined according to the tumor-node-metastasis (TNM) stage (8th American Joint Committee on Cancer) [8]. Pathological classification was based on World Health Organization (WHO) criteria. The detailed information of the patients was listed in Additional file 1: Table S1. The written informed consent was obtained from each patient. This study was approved by the ethics committee on human research of Zhongshan Hospital, Fudan University, and conducted according to the principles of the Declaration of Helsinki.

### Cell lines culture, plasmids, lentiviral vectors with eIF3b and transfection

The human ESCC cell lines EC109 and KYSE510 were purchased from the Institute of Biochemistry and Cell Biology at the Chinese Academy of Science at 2012 and got the authentication from the company. Cells were tested for mycoplasma contamination every 2 months with Mycoplasma Test Kit (YEASEN, CN). Cells were cultured in DMEM medium with 10% fetal bovine serum and 100 IU/ml penicillin/streptomycin in a humidified incubator. The culture condition was 95% air, 5% CO_2_ and 37 °C temperature.

The lentiviral vectors with eIF3b was constructed by Shanghai Genomeditech Company Ltd., (Shanghai, China). The targeted sequences of sh-eIF3b are: 5′-GGAAGCAGATGGAATCGATTC-3′. The ov-eIF3b lentiviral vectors was constructed according to NM_003751.3. The sequence of NC lentiviral vectors was a non-targeted irrelevant sequence. The constructed virus and polybrene were added into the culture medium according to the instruction and co-cultured for 24 h. Forty-eight hours later, puromycin was added to the culture medium to exclude the cells without successful transfection.

Lipofectamine 2000 (Invitrogen, Carlsbad, USA) was employed for transient transfection. The transfection procedure was applied according to the protocol [9]. Predesigned siRNA duplexes were purchased from Biotend Company (Shanghai, China). The sequences of siRNA-TEX9 are 5′-CAGGCUGCAAGUAGUCAAAdTdT-3′ (F) and 5′-UUUGACUACUUGCAGCCUGdTdT-3′ (R). The cells with TEX9 single knockdown (SKD cells) were normal ESCC cells transfected with siRNA-TEX9 sequence. The cells with double knockdown of TEX9 and eIF3b (DKD cells) were sh-eIF3b cells transfected siRNA-TEX9 sequence.withrespectively established. The control (si-Control) group was the cells transfected with a non-targeted irrelevant sequence.

### Quantitative proteomic analysis

For proteome anaylysis, cells were lysed and protein was reduced and then alkylated. Proteins was digested wih Trypsin (Promega, Madison, WI) and peptides were labeled with TMT reagent (Thermo Fisher Scientific, Waltham, MA). eIF3b-depleted cell peptides were labeled with 128 and 129, eIF3b-overexpressed was labeled with 130 and 131, normal control of EC109 cell was labeled with 126 and 127. Twelve fractions were generated by high-pH reverse-phase HPLC. The fractions were resuspended, separated by an EASY-nLC 1000 system connected to an Orbitrap Fusion mass spectrometer (Thermo Fisher Scientific) equipped with an online nano-electrospray ion source. Tandem mass spectra were analyzed using Mascot (Matrix Science, London, UK; version 2.3) and extracted by Proteome Discoverer software (Thermo Fisher Scientific, version 1.4.0.288). Charge state deconvolution and deisotoping were not performed. The percolator algorithm was used to control peptide level false discovery rates (FDR) lower than 1%. Only unique peptides were used for protein quantification and the method of normalization on protein median was used to correct experimental bias.

### Western blot, RNA extraction and RT-qPCR analysis

The total protein was extracted with RIPA lysis buffer and protease inhibitor from these tissues. The Western blot assay were performed in the same way as previously described [10]. The related antibodies which were used to detect the expression of the related protein were listed in the Additional file 2: Table S2. The methods of total RNA extraction and reverse transcribe were performed in the same way with the previous work [9]. RT-qPCR was performed on an ABI 7500 thermocycler (Applied Biosystems, Foster City, CA). The fold change for each target gene relative to the control group was calculated using the ΔΔCt method. The primers of TEX-9 are 5′- GTCTGTGTCTCACGAGAAGCA-3′ (F) and 5′-TAGCTTGTTGAACCACGTCAG-3′ (R). The primers of β-actin are 5′-TGACGTGGACATCCGCAAAG-3′ (F) and 5′-CTGGAAGGTGGACAGCGAGG-3′ (R).

### RNA-immunoprecipitation

RNA-IP was performed with Magna RIP™ RNA-Binding Protein Immunoprecipitation Kit (Millipore, USA), according the manufacture’s protocol. In brief, cell line was lysed by RIP Lysis Buffer with Protease Inhibitor Cocktail and RNase Inhibitor. Cell lysates were incubated with magnetic beads and 5 μg antibody at 4 °C overnight. The samples were placed on the magnetic separator and discard the supernatant. The target protein was confirmed by Western Blot. The immunoprecipitate was washed thoroughly and RNA was purified and further analyzed by RT-PCR and quantitative real-time PCR. The data of quantitative real-time PCR were expressed as percentage relative to the input.

### CCK-8 assay, colony-formation assay and transwell assay

CCK-8 assay and colony-formation assay, which measure the proliferation of cells, were performed as previously described [9, 11]. The OD values, which indicated the cell viability, were detected at the determined hours after transfection. Transwell assay, which measure the migration of cells, was also performed in the same way as previously described [12]. Each experiment was repeated 3 times.

### Flow cytometry for cell apoptosis

At the time point of 48 h after transfection, cells were collected and washed three times with phosphate-buffered saline (PBS) and stained with annexin V and propidium iodide (PI) of Annexin V-FITC/PI Apoptosis Detection Kit (YEASEN, 40302ES, Shanghai, CN). Fluorescence was measured using a FACScan (BD Biosciences, NJ, USA). Annexin V/PI cells were quantified by the frequency of fluorescently labeled cells. Statistical significance was assessed by the two-sample t-test (independent variable).

### Tumor xenograft assay

Four-week-old male nude mice were obtained from Slaccas Company. Totally, 4 × 10^6^ cells of si-Control and si-TEX9 groups were injected subcutaneously into either side of mice posterior flank. Si-Control group was implanted into the left posterior flank and the si-TEX9 group was implanted into the right of the same mouse. Three weeks later, the mice were executed with cervical dislocation and the size of tumors were measured by caliper for statistical analysis.

### Statistical analysis

SPSS 17.0 was applied for statistical analysis. The data drawn from the experiments was noted as “mean ± standard deviation”. Chi-square test or Student t test was applied for two-sample comparisons. Differences among three or more groups were analyzed with a two-way analysis of variance. All the tests at *p*-value < 0.05 were considered as significant difference. The TCGA database was analyzed with “UALCAN” web source [13].

## Results

### TEX9 expression is associated with eIF3b expression and TNM stage in ESCC

**Fig. 1:**
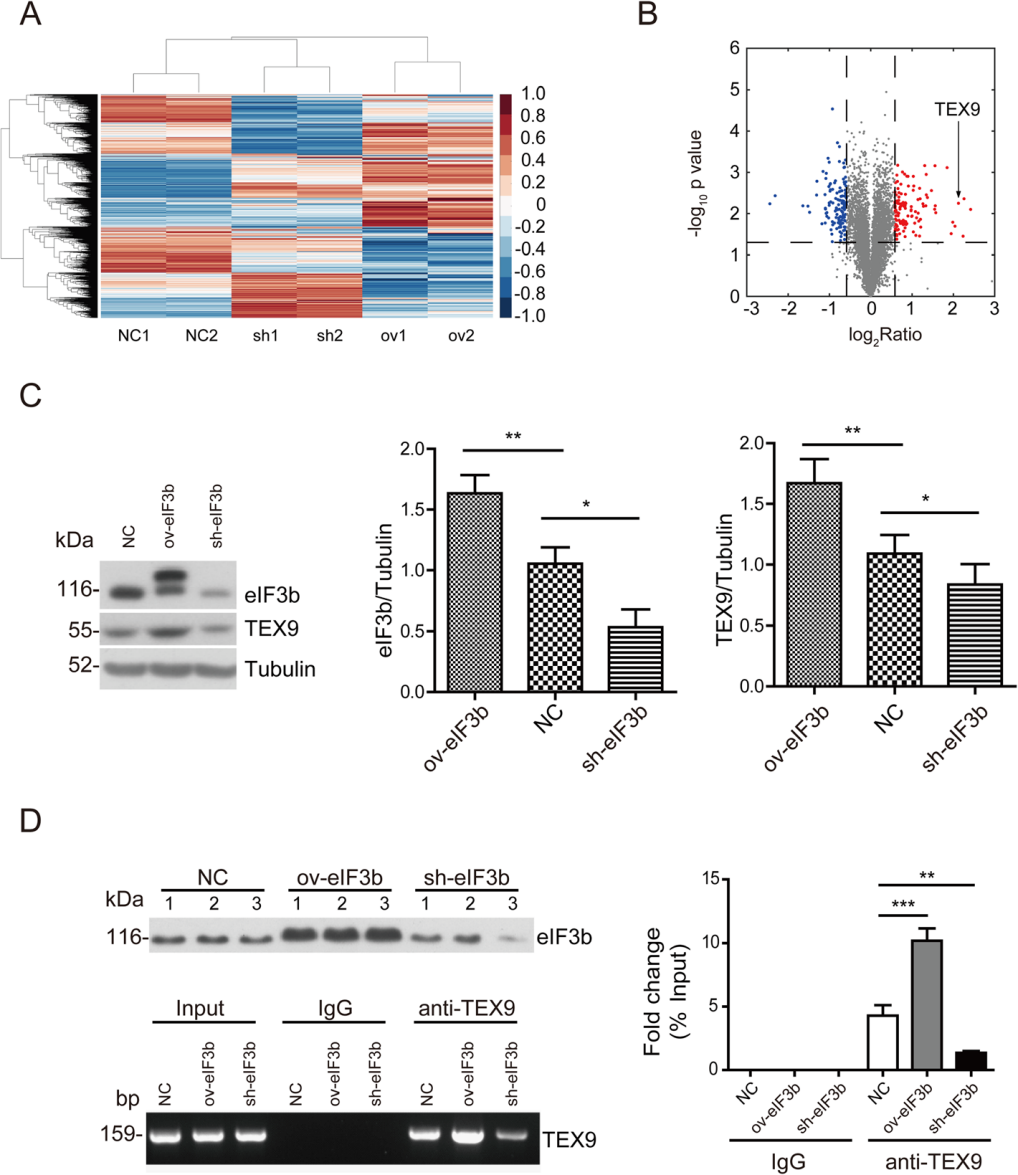
TEX9 is associated with eIF3b expression. **a** Comparsion of proteomics data for eIF3b-depleted (sh1 and sh2, technical replicate), eIF3b-overexpressed (ov1 and ov2, technical replicate) and normal control of EC109 cells (NC1 and NC2, technical replicate). **b** Relative quantification 1.5 fold-changes and *p*-value < 0.05 were selected as being differentially expressed. Significantly up- and down-regulated proteins in eIF3b-overexpressed cells were highlighted in red and blue, respectively. **c** Western blot assay was performed to detect the TEX9 and eIF3b expression in eIF3b-depleted, eIF3b-overexpressed and normal control of EC109 cells. Tubulin was used as an internal reference. **d** RNA-IP was performed to verify that eIF3b can bind to TEX9 mRNA in EC109 cells. The protein level was detected by Western blot assay and the mRNA level was detected by RT-PCR. “NC”, normal control. “sh”, eIF3b-depleted. “ov”, eIF3b-overexpressed. (ns: no significance, *p < 0.05, **p < 0.01, ***p < 0.001)

Quantitative proteomics was performed in eIF3b-depleted, eIF3b-overexpressed and normal control of EC109 cells. The effect of depletion and overexpression was verified (Additional file 3: Figure S1A). The results of quantitative proteomics analysis showed that the upregulated or downregulated proteins in two technical replicates with relative quantification 1.5 fold-changes and *p*-value < 0.05 were selected as being differentially expressed (Fig. 1a and b) and all quantified proteins were listed in Additional file 4: Table S3. The data showed that TEX9 was the only one protein, upregulated (ov1:2.29; ov2:2.04) and downregulated (sh1:0.51; sh2:0.49) in two technical replicates with relative quantification 2 fold-changes. Then, we proved that TEX9 expression was increased significantly in eIF3b-overexpressed EC109 and decreased significantly in eIF3b-depleted EC109 (Fig. 1c), which indicated that Western blot results confirmed quantitative proteomics. Then, RNA-binding protein immunoprecipitation assay was applied to explore whether eIF3b coordinately regulated the rate of mRNA translation of TEX9. The immunoprecipitate mRNA was detected using RT-PCR and RT-qPCR. As shown in Fig. 1d, TEX9 mRNA expression can be detected in the eIF3b binding mRNA, and TEX9 mRNA level was positively correlated with eIF3b protein level. These results indicated that ESCC progression induced by eIF3b may be mediated in part through TEX9.

**Fig. 2:**
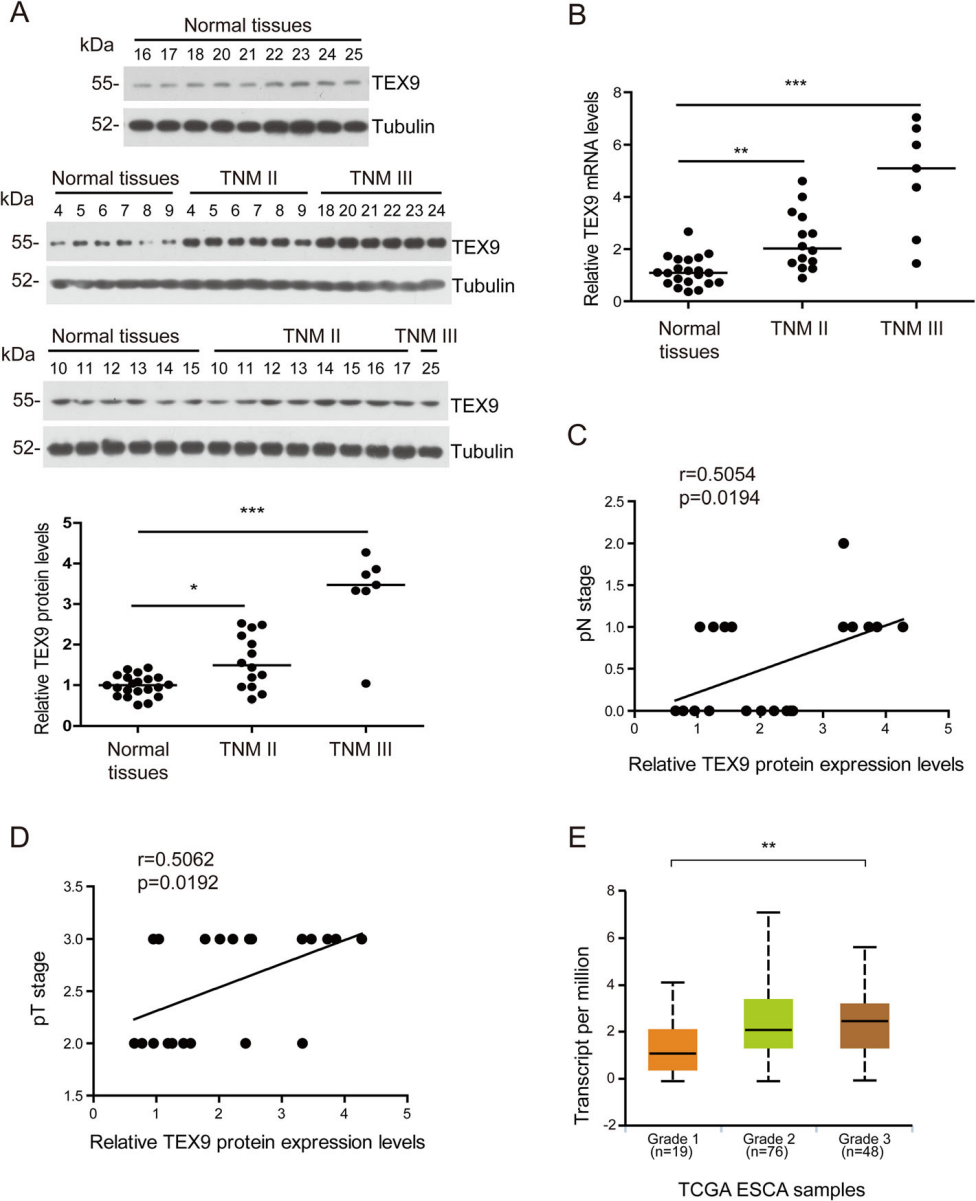
TEX9 expression is associated with TNM stage in ESCC. **a** Western blot assay was performed to detect the TEX9 protein level in normal esophageal tissues and ESCC. Tubulin was used as an internal reference. Densitometry analysis was used to quantitate protein expression with Image J (bottom). **b** RT-qPCR was performed to detect the TEX9 mRNA level in normal esophageal tissues and ESCC. β-actin was used as an internal reference. **c** and **d** Correlations assessed by Spearman’s correlation between protein (Western blot) level of TEX9 and pN stage (**c**) or pT stage (**d**). **e** The TEX9 mRNA expression in each differentiation stage was also analyzed based on TCGA database. “ESCA” stands for esophageal carcinoma in TCGA database. (ns: no significance, *p < 0.05, **p < 0.01, ***p < 0.001)

Western blot and RT-qPCR were performed to detect TEX9 expression in ESCC tissues. The results showed protein and mRNA level of TEX9 was significantly higher in ESCC tissues than that in adjacent normal tissues and had a positive correlation with TNM stage (Fig. 2a and b). We further analyzed the correlation between TEX9 expression level and tumors’ features and found that TEX9 expression was positively correlated with pT, pN stage and the number of metastatic lymph nodes (Fig. 2c, d and Additional file 5: Figure S2). Also, according to the TCGA dataset, TEX9 expression level was positively correlated with the tumor differentiation (Fig. 2e).

### TEX9 binds with eIF3b to promote proliferation and migration, inhibit apoptosis of ESCC cell

**Fig. 3:**
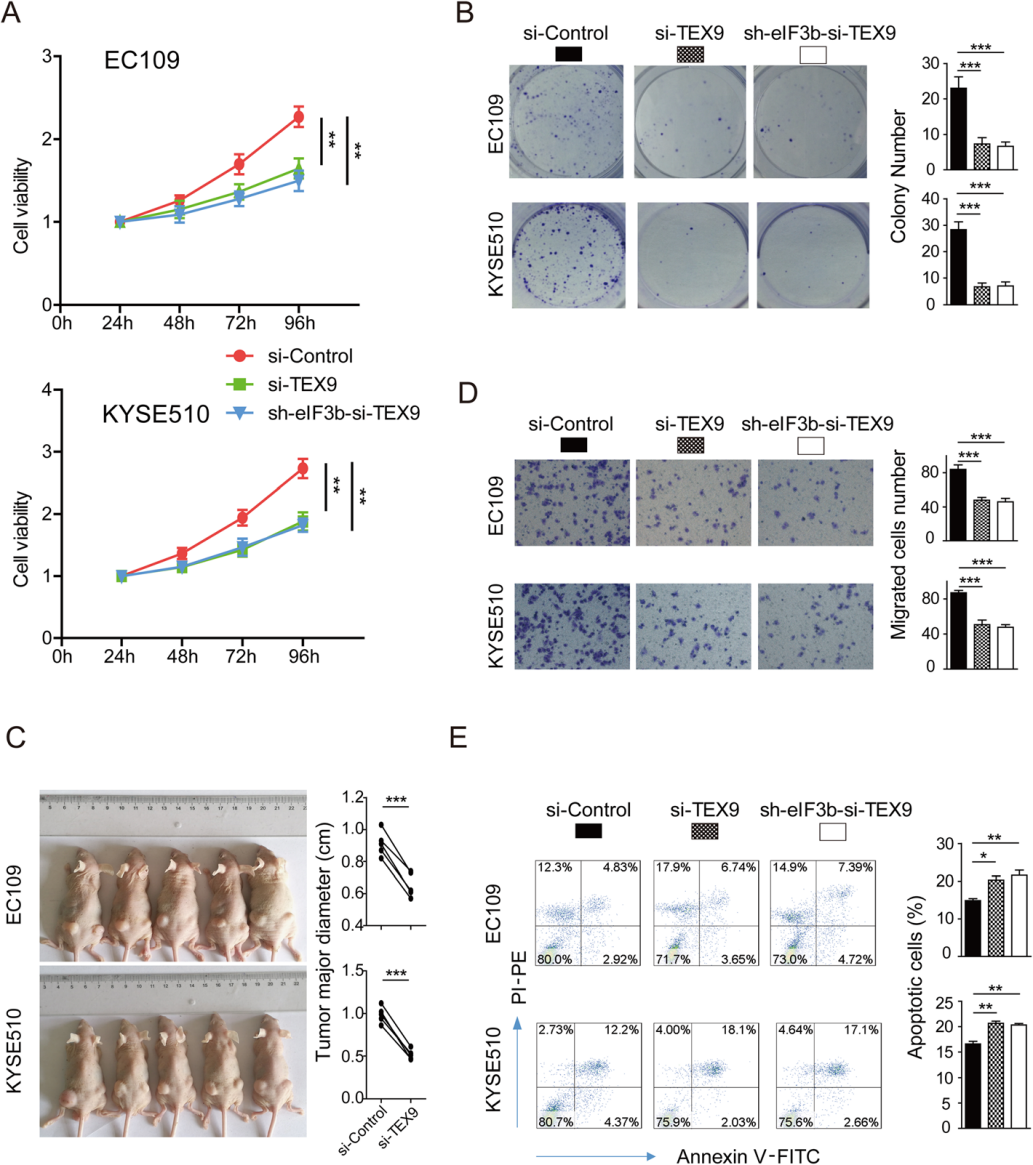
TEX9 binded with eIF3b to promote proliferation and migration, inhibit apoptosis of ESCC cell. **a** The proliferation ability was assessed with CCK-8 assay at 24, 48, 72 and 96 h after transfection. **b** The proliferation ability was measured using colony-formation assay. **c** The migration ability was measured using Transwell assay. **d** The proliferative ability was assessed with tumor xenograft assay and analyzed statistically. Si-Control group was implanted into the left posterior flank and the si-TEX9 group was implanted into the right of the same mouse. **e** The apoptosis of cells was detected with flow cytometry after staining of Annexin V and PI. (ns: no significance, *p < 0.05, **p < 0.01, ***p < 0.001)

Cell proliferation and migration are important components of cancer genesis and metastasis. ESCC cells (EC109 and KYSE510) with single TEX9 knockdown (SKD cell) and double TEX9, eIF3b knockdown (DKD cell) were respectively established (Additional file 3: Figure S1B and C). As shown in Fig. 3a, the proliferation of SKD and DKD cells were significantly inhibited at 72 h after transfection. Both of SKD and DKD cells had lower proliferative ability than control cells using colony-formation assay (Fig. 3b). The tumor xenograft assay also showed that si-Control groups could form significantly larger tumors than the SKD groups did, which indicated that knockdown of TEX9 significantly inhibited the proliferative ability of ESCC cells in vivo (Fig. 3c). SKD and DKD cells showed approximately 50% reduced Transwell cell migration compared with control cells (Fig. 3d). Then, we found that SKD and DKD groups contained more apoptotic cells than control group (Fig. 3e). These results implied that TEX9, synergizing with eIF3b, promoted the proliferation and migration, inhibited the apoptosis of ESCC.

### TEX9 and eIF3b promote the progression of ESCC through the activation of AKT signaling pathway

**Fig. 4:**
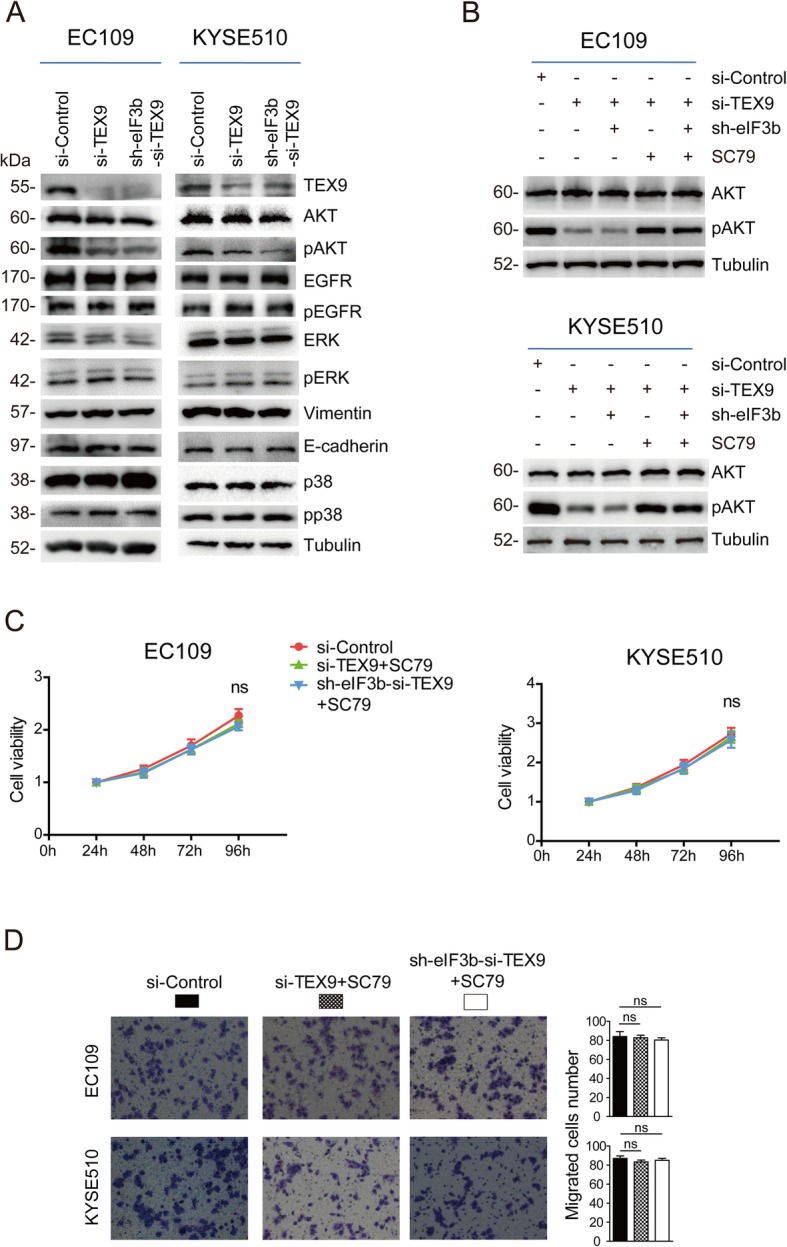
TEX9 and eIF3b promote ESCC progression through the activation of AKT signaling pathway. **a** Western blot assay was performed to analyze the expression difference of the related protein after depletion of eIF3b and TEX9. Tubulin was used as an internal reference. **b** Western blot assay was performed to analyze the AKT and pAKT expression after the treatment of SC79. The cells were starved for 30 min and treated with SC79 (10 μg/mL) for 1 h prior to the extraction of protein. **c** CCK-8 assay was performed to detect the reversed effect on proliferation after the treatment of SC79 for 24 h. **d** Transwell assay was performed to detect the reversed effect on migration after the treatment of SC79 for 24 h. (ns: no significance, *p < 0.05, **p < 0.01, ***p < 0.001)

Limited research focused on TEX9 and cancer progression. In order to explore the mechanism underlying these effects, some classical signaling pathways were considered, including EGFR, AKT, MMP, EMT, etc., which might be the potential downstream pathway of TEX9. Western blot analyses of crucial molecules in those pathways were performed to evaluate their expression as a function of TEX9 and eIF3b depletion. The result revealed that phosphorylated AKT (pAKT) was significantly decreased in both ESCC cells with TEX9 SKD and DKD, however, total AKT protein and the other pathways were not changed (Fig. 4a). In addition, SC79 (a phosphorylation activator of AKT) was applied to determine whether TEX9-promoting ESCC progression was mediated by AKT activation. The pAKT expression was reversed after the treatment of SC79 (Fig. 4b). As shown in Fig. 4c and d, SC79 treatment abolished the inhibitory effects of TEX9 SKD or DKD on proliferation and migration of ESCC cells. Taken together, these data suggested that TEX9, synergizing with eIF3b, promoted the progression of ESCC cells by the activation of AKT signaling pathway.

## Discussion

Several studies have demonstrated that overexpressed eIF3b could promote the progression of malignant cancers such as glioblastoma cells [14],colon cancer [15], osteosarcoma [16] and lung cancer [17]. In addition, we have revealed the tumor-promoting function of eIF3b in ESCC in previous study [6], however, the underlying molecular mechanism remains elusive. In this study, we performed quantitative proteomic analyses and found TEX9 positively correlated with eIF3b expression. Furtherly, we determined that eIF3b binding to TEX9 mRNA in ESCC cells. Through SKD of TEX9 or DKD of TEX9 and eIF3b, we demonstrated that TEX9 could synergize with eIF3b to promote the proliferation and migration, and inhibit the apoptosis of ESCC cells through the activation of AKT signaling pathway.

TEX9 is a member of Testis expressed protein, belonging to cancer/testis antigens (CTA), which are normally expressed only in the testis, except for the expression in early-developing embryos and placentas. When human suffer from cancer, some CTAs expression can be induced in the tumor cells [18]. For the reason that the expression of HLA molecules remains low, testis is not accessible by cytotoxic T lymphocytes (CTLs), so it is called “immunoprivileged organ” [19]. Thus, CTAs have became the promising targets for tumor immunotherapy, including cancer vaccination, adoptive T-cell transfer and immune checkpoint inhibitors [20]. Some Testis expressed protein have been reported to be overexpressed in tumors and accelerate tumor progression. For example, TEX19 exhibited increased expression in bladder cancers and might serve as a potential therapeutic target [21]. TEX19 could drive cell proliferation in colon cancers, possibly mediated via an oncogenic transcript regulation mechanism [22]. Testis expressed protein also played important roles in cancer stem-like cells. For example, TEX10 was upregulated and promoted cancer stem cell properties and chemoresistance in hepatocellular carcinoma [23]. A large number of CTAs showed preferential expression in cancer stem-like cells, including high expression of TEX15 in side population cells [24]. In addition, a rare variant Q1631H in DNA repair gene TEX15 is associated with prostate cancer risk [25] and truncating variants in TEX15 were proved to be potential breast cancer risk factor. Our data demonstrated that TEX9 served as the downstream target, functionally synergizing to promote the proliferation and migration, and inhibited the apoptosis of ESCC cells. In the analysis of mechanism, we revealed that TEX9 and eIF3b promoted the progression of ESCC through the activation of AKT signaling pathway.

## Conclusions

Limited research has revealed that TEX9 participated in tumor progression. In this study, we uncovered its potential correlation with eIF3b in tumor as a CTA. Furtherly, our results provided evidence that eIF3b could bind to TEX9 mRNA, which may form synergized function. They can promote the cells proliferation and migration, and inhibit cells apoptosis of ESCC through the activation of AKT signaling pathway. Hence, eIF3b-TEX9 regulatory axis might offer a novel therapeutic strategy to prevent or postpone the progression of ESCC.

## Additional files

Additional file 1: **Table S1.** The detailed information of the patients' specimen. (XLS 33 kb)

Additional file 2: **Table S2.** The antibodies used in this study. (DOC 38 kb)

Additional file 3: **Figure S1.** The verification of knockdown and overexpression effect. (A) the knockdown and overexpression effect of eIF3b were verified with Western blot in EC109. (B) the knockdown effect of TEX9 was verified with Western blot in EC109 and KYSE510. (C) the double knockdown of eIF3b and TEX9 was verified with Western blot in EC109 and KYSE510. (DOCX 255 kb)

Additional file 4: **Table S3.** The quantified proteins detected with the quantitative proteomics. (XLSX 4526 kb)

Additional file 5: **Figure S2.** The correlations assessed by Spearman's correlation between protein (Western blot) level of TEX9 and the number of metastatic lym nodes. (DOCX 42 kb)

## Data Availability

The information of patients was available in Additional file 1: Table S1. The information of antibodies used in this study was available in Additional file 2: Table S2. The sequence data was available in Additional file 4: Table S3. The other datasets used and/or analysed during the current study are available from the corresponding author on reasonable request.

## Abbreviations

CTA: Cancer/testis antigens
CTLs: Cytotoxic T lymphocytes
DFS: Diseasr-free survival
DKD: Double TEX9 eIF3b knockdown
eIF3b: Eukaryotic translation initiation factor 3 subunit b
ERBB2 or HER2: Erb-b2 receptor tyrosine kinase 2
ESCC: Esophageal squamous cell carcinoma
LC-MS/MS: Liquid chromatography tandem mass spectrometry
OS: overall survival
SKD: Single TEX9 knockdown
TCGA: The Cancer Genome Atlas
TEX9: Testis-expressed protein 9
TNM: Tumor, Node, Metastasis
VEGF: Vascular endothelial growth factor
WHO: World Health Organization
