# Supplementary material for: TEX9 and eIF3b functionally synergize to promote the progression of esophageal squamous cell carcinoma
Source: BMC Cancer. 2019 Sep 3;19:875. doi: 10.1186/s12885-019-6071-9 (PMC6724304; doi:10.1186/s12885-019-6071-9)
Supplement: Supplementary file 2 — Table S2. The antibodies used in this study. (DOC 38 kb) [file 12885_2019_6071_MOESM2_ESM.doc]

**Table S1: The detailed information for the antibodies used for Western blot**

| Antibody | Company | Catalog No. |
| --- | --- | --- |
| EGFR | Epitomics | 2116-S |
| pEGFR | Epitomics | 1727-1 |
| AKT | Cell Signaling Technology | 9272 |
| pAKT | Cell Signaling Technology | 2965P |
| ERK | Cell Signaling Technology | 4695S |
| pERK | Cell Signaling Technology | 4370S |
| E-cadherin | Abcam | Ab1416 |
| Vimentin | Santa Cruz | sc-32322 |
| p38 | Cell Signaling Technology | BA0573 |
| pp38 | Cell Signaling Technology | 9212 |
| β-actin | Proteintech | HRP-60008 |
| β-tubulin | Huabio | 0807-2 |
| TEX9 | ThermoFisher | PA5-24110 |
| Secondary antibody (goat anti-rabbit IgG) | beyotime | A0208 |
| Secondary antibody (goat anti-mouse IgG) | beyotime | A0216 |

“p” in the given proteins refers to phosphorylated.
