# Supplementary material for: TEX9 and eIF3b functionally synergize to promote the progression of esophageal squamous cell carcinoma
Source: BMC Cancer. 2019 Sep 3;19:875. doi: 10.1186/s12885-019-6071-9 (PMC6724304; doi:10.1186/s12885-019-6071-9)
Supplement: Supplementary file 3 — Figure S1. The verification of knockdown and overexpression effect. (A) the knockdown and overexpression effect of eIF3b were verified with Western blot in EC109. (B) the knockdown effect of TEX9 was verified with Western blot in EC109 and KYSE510. (C) the double knockdown of eIF3b and TEX9 was verified with Western blot in EC109 and KYSE510. (DOCX 255 kb) [file 12885_2019_6071_MOESM3_ESM.docx]

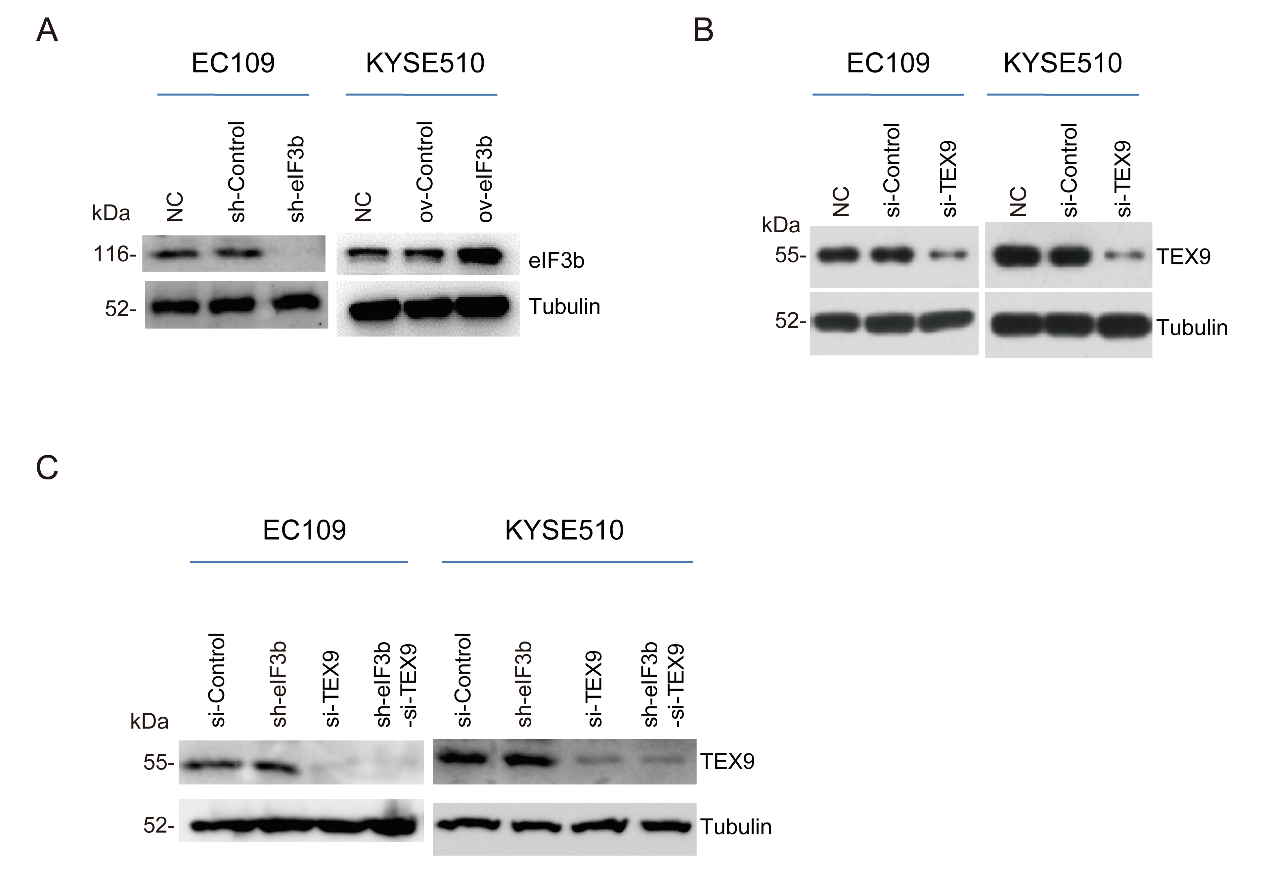


Figure S1

(A) the knockdown and overexpression effect of eIF3b were verified with Western blot in EC109. (B) the knockdown effect of TEX9 was verified with Western blot in EC109 and KYSE510. (C) the double knockdown of eIF3b and TEX9 was verified with Western blot in EC109 and KYSE510.
