# Supplementary material for: TEX9 and eIF3b functionally synergize to promote the progression of esophageal squamous cell carcinoma
Source: BMC Cancer. 2019 Sep 3;19:875. doi: 10.1186/s12885-019-6071-9 (PMC6724304; doi:10.1186/s12885-019-6071-9)
Supplement: Supplementary file 5 — Figure S2. The correlations assessed by Spearman's correlation between protein (Western blot) level of TEX9 and the number of metastatic lym nodes. (DOCX 42 kb) [file 12885_2019_6071_MOESM5_ESM.docx]

Figure S2

Correlations assessed by Spearman's correlation between protein (Western blot) level of TEX9 and the number of metastatic lymph nodes.
